# SB-273005, an antagonist of αvβ3 integrin, reduces the production of Th2 cells and cytokine IL-10 in pregnant mice

**DOI:** 10.3892/etm.2014.1667

**Published:** 2014-04-07

**Authors:** SHAOJUAN WANG, JING YANG, CHONGYANG WANG, QING YANG, XIAOLI ZHOU

**Affiliations:** 1 Department of Reproductive Medical Center, Renmin Hospital of Wuhan University, Wuhan, Hubei 430060, P.R. China; 2 Department of Gynecology, Maternity and Child Healthcare Hospital, Shenzhen, Guangdong 518172, P.R. China

**Keywords:** integrin, ανβ3 antagonist, SB-273005, helper T cells, cytokines

## Abstract

Pregnancy is associated with complex immunoreactions. In the present study, the effect of SB-273005, an antagonist of αvβ3 integrin, on the alterations of T helper (Th) cells and their derived cytokines that occur during pregnancy was investigated in mice. Five non-pregnant mice were used as a negative control. Mice were impregnated by co-housing females and males at a ratio of 2:1 overnight and pregnancy was confirmed by the appearance of vaginal plugs the following morning. Day 1 (D1) pregnant mice were randomly divided into two groups (n=20) and were administered either dimethylsulfoxide (mock treatment) or SB-273005 (3 mg/kg) by gavage at D3, D4 and D5. At D8, the levels of Th1 and Th2 cells and interleukin (IL)-2 and IL-10 in the spleen and peripheral blood were determined using flow cytometry and enzyme-linked immunosorbent assay. Pregnancy significantly increased the ratio of Th2:Th1 cells in the spleen compared with that in non-pregnant mice (P<0.01). However, this increase was significantly reduced by SB-273005 (P<0.001). Furthermore, whilst pregnancy decreased Th1 cell-produced IL-2 levels and increased Th2 cell-derived IL-10 levels, SB-273005 reversed both processes (P<0.05 for IL-2; P<0.01 for IL-10). The results from the present study demonstrated that pregnancy induces changes in the spleen, including a reduction of IL-2 and an increase in IL-10 production by Th1 and Th2 cells, respectively, as well as an upregulation of the Th2:Th1 ratio in the spleen. These immunological changes are reversed by SB-273005, indicating an important role for αvβ3 integrin in mediating these immunological alterations.

## Introduction

Implantation of the zygote to the endometrium is dependent on the interaction between the cell and the extracellular matrix. Integrins are of particular importance as they are known to bind to the extracellular matrix. In 1989, Hynes *et al* (1) identified that integrins are a family of cell adhesion molecules. Integrins are transmembrane glycoprotein receptors that are widely distributed on cell surfaces (1). Individual integrins consist of an α and a β chain. At present, 18 α and eight β chains have been discovered and different combinations of these chains produce 24 different integrins in mammals (2). The ανβ3 integrin is an important member of the integrin family and has a critical role in the adhesion of vascular endothelial cells and tumor cells during angiogenesis and tumor metastasis (3–7). SB-273005, (4S)-2,3,4,5-tetrahydro-8-[2-[6-(methylamino)-2-pyridinyl] ethoxy]-3-oxo-2-(2,2,2-trifluoroethyl)-1H-2-benzazepine-4-acetic acid, is an ανβ3 integrin antagonist (8). SB-273005 is a benzalkonium-derived compound that was synthesized by Miller *et al* (9) at SmithKline Beecham.

SB-273005 is a candidate drug for the therapeutic treatment of patients with osteoporosis (10). It has been found to protect bone and soft tissue in a mouse model of arthritis (10). Furthermore, SB-273005 has also been shown to inhibit the adhesion and migration of αvβ3-positive breast cancer cells, which makes SB-273005 a candidate for a therapeutic cancer drug for the prevention of tumor-associated angiogenesis (11). In addition, Wang *et al* (12) previously demonstrated that SB-273005 affects the expression of the ανβ3 integrin in the reproductive system.

Implantation is regarded as a successful allograft since it relies on the establishment and consolidation of an immune balance between the mother and fetus (13). Implantation failure may be caused by multiple factors, including an imbalance in immunological reactions (14). It has been previously reported that variations in immune responses and immunomodulatory mechanisms between the mother and fetus may influence the dynamic balance between T helper type 1 (Th1) and Th2 cells (15–19).

In a previous study, we demonstrated that SB-273005 has a negative effect on implantation, and that this anti-implantation activity was due to SB-273005-derived reduction of ανβ3 integrin levels in the mouse decidual cells (12). SB-273005 may inhibit the implantation of embryos, promote embryo degeneration and reduce the blastocyst rate (12), resulting in significantly lower conception efficiency. However, the underlying mechanism by which SB-273005 modulates pregnancy-associated immunoreactions has yet to be elucidated.

To investigate these mechanisms, changes in the levels of Th1 and Th2 at implantation were investigated in the present study. The percentages of Th cells and the levels of cytokines in the peripheral blood and spleen were analyzed using flow cytometry and enzyme-linked immunosorbent assay (ELISA), respectively. The hypothesis that implantation is associated with changes in the spleen regarding Th1 and Th2 cells and cell-derived interleukin (IL)2 and IL-10 was examined. The potential role of αvβ3 integrin in these changes was investigated by the inhibition of αvβ3 with SB-273005.

## Materials and methods

### Experimental animals

Outbred pathogen-free grade Kunming mice (between 6 and 8 weeks old; weighing 25–30 g) were purchased from the Laboratory Animal Center of Sun Yat-sen University [Guangzhou, China; certificate of conformity, 0080353; license no. SCXK (Yue) 2009–0011]. The mice were kept at 25°C with a light cycle of 12 h on and 12 h off and free access to food and water. All animal experiments were performed at Sun Yat-sen University according to experimental protocols approved by the University Animal Research Ethics Board.

### Reagents and instruments

SB-273005 was obtained from GlaxoSmithKline (London, UK); dimethylsulfoxide (DMSO) was purchased from Sigma (St. Louis, MO, USA); cluster of differentiation (CD)3-Per, CD8-fluorescein isothiocyanate, CD8 negative control, interferon (IFN)-γ-phycoerythrin (PE), CD16^+^CD56-PE, mouse-immunoglobulin G (IgG)1-Per, mouse-IgG1-PE, anti-human IL-4-PE, and permeabilization solution were purchased from BD Biosciences (Franklin Lakes, NJ, USA); and trypsin and culture solutions were purchased from Gibco-BRL (Carlsbad, CA, USA). All other reagents were purchased from Sigma.

### Establishment of a pregnancy mouse model

Female mice were impregnated by one-time co-caging with males at a 2:1 ratio overnight. Mice were examined for vaginal plugs the following morning. Mice with clearly visible vaginal plugs were recorded as being day 1 (D1) pregnant. The pregnant mice were randomly divided into two groups (n=20 in each group). The mice in the pregnancy drug group were continuously administered SB-273005 (dissolved in DMSO) at 3 mg/kg (0.1 ml/mouse) on D3, D4 and D5 by gavage, whilst mice in the normal pregnancy group received DMSO only. Blood was collected on D8 in the presence of heparin to prevent coagulation.

### Separation of lymphocytes from peripheral blood

Lymphocytes in the peripheral blood were separated by density gradient centrifugation using lymphocyte separation medium in accordance with the manufacturer’s instructions (Tianjin Hao Yang Biological Products Co., Ltd., Tianjin, China). A total of 1 ml heparin anti-coagulated whole blood was diluted with 1 ml Hank’s solution. The solution was then slowly dripped into double volume of lymphocyte separation medium along the tube wall using a dropper, followed by centrifugation at 544 × g using a swing bucket rotor (15 cm radius) for 15 min. The centrifugation force separated materials into four layers, with the first, second and third layer being the plasma, circular milky lymphocytes and transparent separation medium, respectively. The cells in the second layer were collected, and 5 ml phosphate-buffered saline (PBS) was added to the cells. The cell solution was thoroughly mixed and centrifuged at 371 × g for 10 min. Cells were then washed twice and resuspended with modified RPMI-1640 medium (containing penicillin, 100 U/ml, and streptomycin, 100 U/ml) supplemented with 10% fetal bovine serum (FBS) to a density of 1×10^7^/ml for culture.

### Preparation of the spleen lymphocyte suspension

Preparation of lymphocytes from the spleen was performed as previously described (20). Briefly, the spleen was harvested from the abdominal cavity under sterile conditions and sectioned into small pieces in a small volume of PBS. The spleen was then ground and filtered through a steel mesh (100 mesh) and a nylon mesh (200 mesh), and rinsed with Hank’s solution. A single-cell suspension was then prepared by centrifugation at 165 × g for 5 min, followed by the addition of 10 ml erythrocyte lysis buffer to the cell pellet. The cells were further incubated for 4–5 min to allow the rupture of red blood cells, and then centrifuged at 165 × g for 5 min. The cells were then washed Hank’s solution 2 or 3 times prior to being resuspended in medium (5×10^6^/ml of culture).

### Culture of lymphocytes

Lymphocytes were obtained from the peripheral blood and spleen and had a viability of >95% according to the results of trypan blue staining. Cells were seeded into a 24-well culture plate containing 0.1 μl phorbol ester and incubated in a tissue culture incubator at 37°C at 5% CO_2_. After 2 h, non-adherent cells were removed via a medium change. Adhesive cell growth was detected after 4–6 h of culture using an inverted microscope. Cells were then re-seeded at 1×10^6^/ml following trypsinization for culture until confluence (~3–4 days), and the medium was changed every other day.

### Analysis of cells using flow cytometry (BD FACSAria™ Fusion, Becton Dickinson and Company, Franklin Lakes, NJ, USA)

A total of 2.5 μl anti-CD4 was added to a 100-μl cell solution, and the cells were incubated for 15 min at room temperature. Cold fixation solution (500 μl) was added and the cells were further incubated for 10–20 min at room temperature. A total of 1 ml FBS was then added to the cell solution, and the cells were centrifuged at 323 × g for 10 min. The cells were washed with FBS and 1 ml permeabilization solution was added prior to incubation for a further 15 min at room temperature. Cells were then collected via centrifugation at 323 × g for 10 min. Then, 1 μl IFN-γ-FITC and 2.5 μl IL-4 was added and the cells were incubated at room temperature for 30 min, followed by two washes with 1 ml FBS via centrifugation at 323 × g for 10 min. A total of 300 μl FBS was then added prior to the flow cytometric analysis. All procedures were performed under light-proof conditions.

### Flow cytometry procedure

The levels of cytokines in 5,000 lymphocytes were evaluated by flow cytometric analysis. The data were analyzed using CellQuest™ software (BD biosciences).

### Detection of cytokines

Lymphocyte cells obtained from the peripheral blood and spleen were incubated at 37°C in a tissue culture incubator containing 5% CO_2_ for 72 h. The cell culture medium was collected via the removal of cellular debris by centrifugation at 672 × g for 20 min. The levels of the cytokines IL-2 and IL-10 were determined using a double-antibody sandwich ELISA. The cytokine activity in the supernatant was determined in accordance with the manufacturer’s instructions.

### Statistical analysis

Statistical analysis was performed using SPSS software, version 13 (SPSS, Inc., Chicago, IL, USA). Data were analyzed using a 2-tailed Student’s t-test. P<0.05 was considered to indicate a statistically significant difference.

## Results

### Pregnancy induces alterations in the levels of Th1 and Th2 cells in the spleen

**Figure 1:**
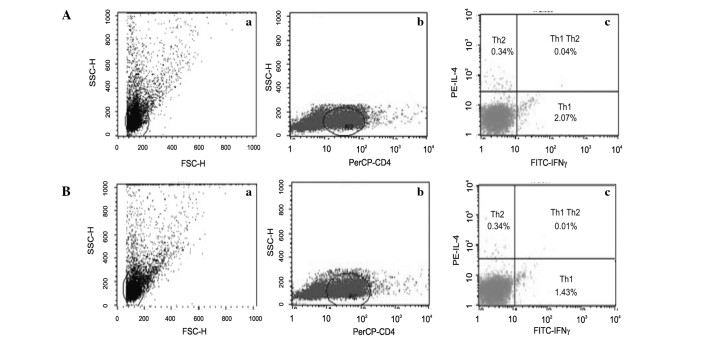
Isolation of lymphocytes from the peripheral blood and spleen. The gated cells in (Aa) and (Ba) represent lymphocytes isolated from the peripheral blood and spleen, respectively. The cells gated in (Ab) and (Bb) are the Th1 and Th2 lymphocytes isolated from the peripheral blood and spleen, respectively. The percentages of Th2 cells in lymphocytes isolated from the peripheral blood (Ac) and spleen (Bc) were identified using flow cytometric-based immunofluorescence staining for IL-4 and the corresponding Th1 cells were marked with anti-IFNγ (Ac and Bc). Th, T helper; IL, interleukin; INF, interferon,

Immunological reactions are a critical factor that determines the success of zygote implantation (20). To investigate the changes in the number of lymphocytes as a result of pregnancy, lymphocytes were isolated from the peripheral blood (Fig. 1Aa) and the spleen (Fig. 1Ba). Among these lymphocytes, the Th1 and Th2 populations were identified (Fig. 1Ab and 1Bb). The percentage of Th2 cells remained consistent (0.34%) in lymphocytes isolated from spleen and peripheral blood; however, the percentage of Th1 cells was slightly higher in peripheral blood lymphocytes compared with spleen lymphocytes (Fig. 1Ac and 1Bc).

**Figure 2:**
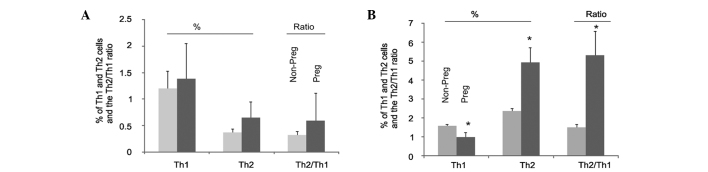
Pregnancy induces specific alterations in Th1 and the Th2 cell populations. Th1 and Th2 cells in (A) the peripheral blood and (B) the spleen in non-pregnant and pregnant mice were quantified. Results are presented as the mean ± standard deviation. The ratios of Th2:Th1 cells are also included. ^*^P<0.05, compared with non-pregnant mice (2-tailed Student’s t-test). Data were derived from 20 non-pregnant and 20 pregnant mice. Th, T helper.

Changes in Th1 and Th2 cells in the spleen and peripheral blood were then investigated during pregnancy. The percentages of Th1 and Th2 cells in the peripheral blood increased in pregnant mice compared with those in non-pregnant mice; however, the increase was not significant (Fig. 2A). These results are consistent with the finding that the ratio of Th2:Th1 cells in the peripheral blood in non-pregnant mice was not significantly different from that in pregnant animals (Fig. 2A). By contrast, analysis of spleen lymphocytes revealed that there was a significant reduction in the percentage of Th1 cells in pregnant mice compared with that in non-pregnant mice (Fig. 2B), with a concomitant increase of the percentage of spleen Th2 cells (Fig. 2B). As a result of these changes, the ratio of Th2:Th1 in the spleen lymphocytes was markedly increased in pregnant mice compared with that in non-pregnant mice (Fig. 2B). In combination, these results demonstrate that there are specific changes in the levels of Th1 and Th2 cells in the spleens of pregnant mice.

### Pregnancy decreases IL-2 expression and increases IL-10 expression

To further investigate the changes in Th1 and Th2 cells during pregnancy, the expression of IL2 and IL10, the specific products of Th1 and Th2 cells, was analyzed (24). The presence of IL-2 and IL-10 in the peripheral blood and the spleen was analyzed using an ELISA. The levels of IL-2 in the peripheral blood were significantly reduced in pregnant mice compared with those in non-pregnant mice, whereas a significant increase in IL-10 levels was observed in the pregnant mice (Table I). The changes in IL-2 and IL-10 levels in the spleen were similar to the results in the peripheral blood (Table I). In the pregnant mice compared with the non-pregnant mice, the level of IL-2 was reduced by 53%, whilst the level of IL-10 was increased by 51% in the spleen (Table I). However, the changes in IL-2 and IL-10 levels observed in the spleen were greater compared with the changes observed in the peripheral blood (Table I). In combination, these results not only demonstrate that there is a pregnancy-associated reduction in IL-2 and an increase in IL-10 levels, but also support the dynamic changes in Th2 versus Th1 cells in the spleens of pregnant mice (Fig. 2).

### ανβ3 integrin has an important role in pregnancy-associated changes in Th1 and Th2 cells in the spleen

**Figure 3:**
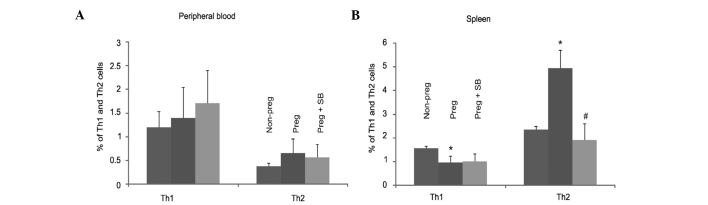
SB-273005 attenuates pregnancy-induced changes in Th1 and Th2 lymphocytes. Th1 and Th2 cells in (A) the peripheral blood and (B) the spleen were analyzed in 20 non-pregnant mice and 20 pregnant mice not treated with SB-273005, and 20 pregnant mice treated with SB-273005. The results are presented as the mean ± standard deviation. ^*^P<0.05, compared with non-pregnant mice (2-tailed Student’s t-test); ^#^P<0.05, compared with pregnant mice (2-tailed Student’s t-test). Th, T helper; SB-273005, (4S)-2,3,4,5-tetrahydro-8-[2-[6-(methylamino)-2-pyridinyl] ethoxy]-3-oxo-2-(2,2,2-trifluoroethyl)-1H-2-benzazepine-4-acetic acid.

ανβ3 integrin has an important role in the implantation of the zygote (21), which suggests that ανβ3 integrin may also have a role in pregnancy-associated changes in Th1/Th2 cells. To investigate this, pregnant mice were either mock-treated or treated with an ανβ3 integrin antagonist reagent (SB-273005) and the levels of Th1 and Th2 cells were then analyzed. Consistent with the findings that there were no changes in either Th1 or Th2 cells in the peripheral blood during pregnancy (Fig. 2A), inhibition of the ανβ3 function by SB-273005 did not significantly alter either cell population (Fig. 3A). Despite the fact that the spleen levels of Th1 cells were reduced in pregnant mice (Fig. 2B), SB-273005 did not alter the levels of Th1 cells in pregnant mice (Fig. 3B). However, inhibition of ανβ3 by SB-273005 markedly reduced the levels of pregnancy-upregulated Th2 cells to the level observed in non-pregnant mice (Fig. 3B). In combination, these results support the hypothesis that ανβ3 integrin has a critical role in the alteration of Th1 and Th2 cells in the spleens of pregnant mice.

### ανβ3 integrin contributes to changes in IL-2 and IL-10 levels during pregnancy

**Figure 4:**
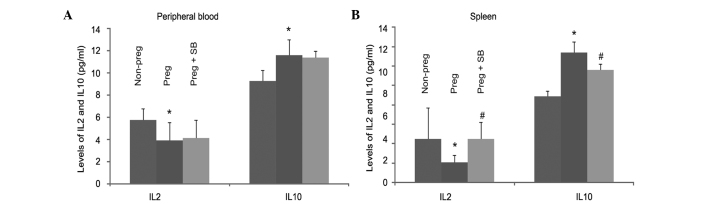
SB-273005 inhibits the pregnancy-induced changes of IL-2 and IL-10. The levels of IL-2 and IL-10 in (A) the peripheral blood and (B) the spleen were measured using an ELISA in 20 non-pregnant mice and 20 pregnant mice not treated with SB-273005, and 20 pregnant mice treated with SB-273005. The results are presented as the mean ± standard deviation. ^*^P<0.05, compared with non-pregnant mice (2-tailed Student’s t-test); ^#^P<0.05, compared with pregnant mice (2-tailed Student’s t-test). IL, interleukin; SB-273005, (4S)-2,3,4,5-tetrahydro-8-[2-[6-(methylamino)-2-pyridinyl] ethoxy]-3-oxo-2-(2,2,2-trifluoro ethyl)-1H-2-benzazepine-4-acetic acid; ELISA, enzyme-linked immunosorbent assay.

Since ανβ3 integrin was found to have a role in pregnancy-associated changes of Th1/Th2 cells (Fig. 3), the role of ανβ3 integrin in the alterations of IL-2 and IL-10 in pregnant mice was investigated. The levels of IL-2 and IL-10 in the peripheral blood and spleen were measured in pregnant mice in the presence and absence of SB-273005. A reduction in the level of IL-2 in pregnant mice was observed in the peripheral blood and the spleen (Table I; Fig. 4), and treatment with SB-273005 significantly inhibited pregnancy-induced reduction of IL-2 in the spleen (Fig. 4). Similarly, pregnancy-induced upregulation of IL-10 was significantly reduced by SB-273005 in the spleen (Fig. 4). In combination, these results demonstrate the important role of the ανβ3 integrin in pregnancy-associated alterations of IL-2 and IL-10. These results also indicate that IL-2 and IL-10 in the spleen are produced primarily by Th1 and Th2 lymphocytes, respectively.

## Discussion

SB-273005 has been previously shown to inhibit embryo implantation, promote embryo degeneration and decrease the blastocyst formation rate (12). As a result, SB-273005 significantly reduces the rate of conception. However, the mechanisms governing SB-273005-derived inhibition of embryo implantation remain unclear. These were investigated in the present study and the results indicate that one of the mechanisms involved is interference in the unique immunoreactions required for embryo implantation.

Implantation is considered a successful allograft and it is associated with complex immunological changes. Li *et al* (22) previously demonstrated that overexpression of Th1 cytokines results in miscarriage. This is consistent with the results from Jin *et al* (23), which showed that IL-2 and IFN-γ were highly expressed in the maternal-fetal interface during human miscarriage and the expression levels of IL-4 and IL-10 were concordantly lower in the interface during miscarriage (23). IL-2/IFN-γ and IL-4/IL-10 are secreted by Th1 and Th2 lymphocytes, respectively (24); therefore, the results from the previous studies support a shift from Th1 to Th2 signaling events during implantation; and preventing this shift is associated with miscarriage.

The results from the present study provide additional support for the downregulation of Th1 signaling and the upregulation of Th2 signaling during embryo implantation. Compared with non-pregnant mice, a change in the Th1 and Th2 cell populations was observed in the spleen of pregnant mice on Day 8 of pregnancy, when zygote implantation takes place. Specifically, a reduction in the percentage of Th1 cells and a concordant increase in the percentage of Th2 cells was detected in the spleen of D8 pregnant mice (Fig. 2B). To further support this shift, the levels of Th1 cell-produced IL-2 decreased, while those of Th2 cell-derived IL-10 increased (Table I). The results from the present study support the use of Th2 signaling over Th1-mediated immunoreactions during zygote implantation, and this is consistent with the idea that elevation of Th2 signaling with concordant inhibition of the Th1 pathway induces immune tolerance, a condition critical for pregnancy (25). The inability to downregulate Th1 cells and cytokines is likely to cause a miscarriage (26).

The mechanisms responsible for the unique alterations of Th1- and Th2-mediated immunoreactions during pregnancy are complex; however, in the present study it was demonstrated that the ανβ3 integrin contributes to these changes. This is based on the observation that SB-273005, a well-established antagonist of ανβ3 (12), attenuated the pregnancy-associated changes in the levels of Th1 and Th2 and their derived cytokines. In particular, SB-273005 increased the levels of Th1 cells and Th1 cell-produced IL2, and decreased the levels of Th2 cells and Th2 cell-derived IL-10 in pregnant mice (Table I). This suggests that SB-273005 may inhibit implantation by reversing the increase in the Th2 to Th1 ratio. Inhibition of Th2 affects the distribution of the associated cytokines, and as a result, a disturbance of immune tolerance may occur, leading to immune reactions and ultimately miscarriage.

The immune balance between the mother and fetus is critical for implantation (27–29). It has previously been shown that this immune tolerance is largely achieved by downregulating Th1 and upregulating Th2-mediated immunoreactions (30), and this is supported by the results of the present study. Furthermore, it was demonstrated in the present study that ανβ3 integrin has a critical role in achieving immune tolerance. However, the detailed mechanisms by which the ανβ3 integrin contributes to reduced Th1 and elevated Th2 signaling require further investigation.

## Figures and Tables

**Table I:** Pregnancy causes changes in interleukin-2 and interleukin-10 levels.

Parameter	Number of mice	Interleukin-2	Change (%)^a^	Interleukin-10	Change (%)^a^
Peripheral blood					
Non-pregnant	20	5.78±0.88		9.29±0.65	
Pregnant	20	3.91±1.63^b^	−32.3	11.61±1.38^b^	+25.0
Spleen					
Non-pregnant	20	4.48±3.19		8.84±0.59	
Pregnant	20	2.09±0.74^c^	−53.3	13.36±1.16^b^	+51.1

a The magnitude of changes associated with pregnancy is calculated by the pregnancy level (non-pregnant or pregnant); − and + show decrease and increase in pregnant animals, respectively.

b P<0.05, compared with non-pregnant mice;

c P=0.001, compared with non-pregnant mice.

## Abbreviations

DMSO: dimethylsulfoxide
FBS: fetal bovine serum
FCM: flow cytometer
IL-2: interleukin-2
IL-10: interleukin-10
PBS: phosphate-buffered saline
Th: T helper cells
SB-273005: (4S)-2,3,4,5-tetrahydro-8-[2-[6-(methylamino)-2-pyridinyl]ethoxy]-3-oxo-2-(2,2,2-trifluoroethyl)-1H-2-benzazepine-4-acetic acid
